# An investigation into the prevalence of cognitive impairment and the performance of older adults in Guilan province

**DOI:** 10.25122/jml-2018-0017

**Published:** 2018

**Authors:** Robabeh Soleimani, Somayeh Shokrgozar, Mahnaz Fallahi, Hashem Kafi, Maryam Kiani

**Affiliations:** 1. Psychiatry Department, Kavosh Cognitive Behaviour Sciences and Addiction Research Center, Shafa educational – remedial Hospital, Associate Professor, Guilan University of Medical Sciences, Rasht, Iran; 2. Psychiatry Department, Kavosh Cognitive Behaviour Sciences and Addiction Research Center, Shafa educational – remedial Hospital, Assistant Professor, Guilan University of Medical Sciences, Rasht, Iran; 3. Vice-chancellor for health, Guilan University of Medical Sciences, Rasht, Iran; 4. Guilan University of Medical Sciences, Rasht, Iran

**Keywords:** Elderly, Cognitive impairment, Daily life functioning

## Abstract

**Introduction:** The escalating rate of old people with a functional impairment in Iran and the weakness of the family support due to the diminishing of family size have increased the demand for long-term care for the elderly with cognitive impairment (CI).

**Objective:** The purpose of this research is to explore the frequency of cognitive impairment in the elderly and its association with their daily functional impairment and disability.

**Method:** This is a cross-sectional and descriptive-analytic study conducted in 2016-2017. The study sample consisted of 393 elderly people who were 60 years old or older who live in of Guilan different counties. Samples were selected by using multi-stage cluster sampling. Subsequently, data were analyzed by using the Chi-square test and correlation and regression analysis conducted in SPSS 22.

**Results:** It was observed that 4.3, 28.6, and 37% of the subjects suffered from severe, moderate, and mild cognitive impairment, respectively. Cognitive impairment had a significant relationship with daily functioning and activities requiring special tools. Moreover, cognitive impairment in women, people with low education, and those over 70 years old was more common, and the difference between them was significant (p <0.001). Also, disability was significantly greater in the elderly with cognitive impairment.

**Conclusion:** Many old people need to be cared for after the appearance of cognitive impairment. Therefore, appropriate screening of cognitive impairments is conducive to early diagnosis and prevention of executive functioning problems.

## Introduction

Old age depends not only on the chronological age of people, but also on their physical and mental abilities, socioeconomic resources, educational background, and previous life experiences because age alone cannot be a determining factor in defining old age [1]. The United Nations has not yet proposed a clear benchmark for determining old age, but as a rule of thumb, people aged 60 or over are considered elderly [2]. Today, old age and gerontology have gained much more attention since the improved health conditions and increased life expectancy in the world have resulted in the boosting of the elderly population [3]. Currently, the world’s population is around 7 billion, and 893 million are elderly. It is estimated that this number will rise in the coming years, and a significant portion of this population will live in developing countries such as Iran [4].

Although some degrees of memory weakness are normal at an older age, the term dementia is used to denote memory decline and forgetfulness among this age group. Since symptoms appear gradually, they may not attract attention for a long time, and thus it may be mistakenly imagined that these behaviors are part of the process of aging. Consequently, people do not pay attention to these symptoms and they do not recognize that such a disorder does exist. Researchers today have found that like other conditions such as heart problems and cancer, Alzheimer’s disease develops over decades and is influenced by lifestyle. Therefore, factors such as cholesterol, high blood pressure, obesity, depression, education, nutrition, sleep, mental status, and physical as well as social activities affect it. However, many simple day-to-day activities may protect a person against memory loss caused by Alzheimer’s disease. Cognitive impairments are among the most prevalent diseases in the elderly, and in more than half of the old people, mild cognitive impairment progresses to dementia within five years [5]. Hence, punctual screening of cognitive impairment may be helpful in early detection and timely treatment. Cognitive impairment is an aging-related disease, which can result in various health problems, including disability and death. It has been reported that the prevalence of cognitive impairment is greater than 40% among elderly individuals [6]. Cognitive impairment is not only highly prevalent but has a great impact on the quality of life, thus imposing a substantial socioeconomic burden. Cognitive impairment is now a significant public health concern in Mainland China [7], and identifying potential risk factors of cognitive impairment is fundamental for developing preventive strategies.

The most common symptom in the early stages of dementia is the weakening of short-term memory. On the other hand, in the early stages of dementia, one of the most sensitive symptoms which are most likely to reveal some changes is the disability of the individual to use essential equipment in everyday activities. Among these activities, changes in one’s capability to manage financial affairs and control his/her medication are the first to emerge. Gradually, as the patient approaches moderate dementia, disability affects simple everyday activities so much that the individual becomes entirely dependent. As people grow older, their quality of life increasingly becomes dependent on the ability to maintain their autonomy. While life expectancy is an important indicator of population aging, the question of how long people can expect a life without disabilities has remained a special concern for the future elderly community [8-9].

Early research in cognitive impairment primarily studied elders aged between 65 and 80 years (10), and a few even excluded those aged over 80 years old [11]. However, lately, as the highest-risk population for cognitive impairment and the fastest-growing population segment, the elderly aged 80 years or over has been increasingly suggested to be studied as an independent population [12].

Working on a qualitative study on seniors with a mild cognitive impairment that had referred to an outpatient geriatric hospital, De Vriendt et al. assessed the effect of cognitive impairment on the activities of daily living (ADL). These activities had been classified in terms of difficulty, complexity, and vulnerability to three groups of basic ADL, instrumental ADL (IADL) and advanced ADL. Data showed that advanced ADL plays an essential role in the diagnosis of cognitive impairment, and consequently, it is essential to integrate it into cognitive impairment assessment tools [13]. In a retrospective study on 600 seniors aged 65 or over who lived in the society, Millán-Calenti et al. examined the role of cognitive impairment in predicting the functional dependency of older adults. In the present research, using a logistic regression model, the relationship between cognitive impairment and age, level of education, and dependency in ADL and IADL was evaluated under different health and socio-demographic circumstances. Data indicate that the relationship between cognitive impairment and the level of dependency in ADL and IADL has a progressive nature so that with the increase in age, the incidence of these two types of dependence rises too [14]. Studies suggest that in the future, the senior population will continue to grow at a faster pace and Iran will encounter an unprecedented geriatric population. The increase in age causes physical and cognitive deficits in the elderly and threatens their health [5].

The aim of this study was to describe the general prevalence of cognitive impairment in elderly patients living in the Guilan province and to investigate the relationship between advanced age and cognitive impairment, daily functioning, activities requiring special tools, and disability.

## Methodology

This is a cross-sectional, descriptive-analytic study. The study population included the elderly aged 60 years or over who live in Guilan province and had received health services in one of the health centers of the region. These people filled out the questionnaires with the help of the research assistants and mental health experts. The assistants had been trained on how they should interview the subjects, complete the questionnaire, and score them. Finally, the questionnaire was completed within three months of 2016. In order to determine the study population, this group was classified according to gender and age. The enrolled subjects were selected using random sampling. According to the census of 2016, 121.283 men were over the age of 60, and 121.567 women were over 60 years old in urban and rural areas, respectively. Totally, 242.850 individuals were from urban and rural areas. Eventually, a total of 393 seniors aged over 60 were surveyed.

Cluster sampling was carried out randomly in several steps. Thus, first, based on the percentage of the elderly population with respect to the whole population, 201 women and 192 men were selected. On the basis of random sampling, the sample size was estimated at 393 people.

After data were collected and codified, they were imported into the SPSS 22 software. Subsequently, using descriptive statistics (mean, median, standard deviation, number, and percentage) and analytical statistics (Spearman correlation coefficient test, Chi-square test, and regression analysis), the authors analyzed the data. After the researchers obtained permission from the Ethics Committee of Guilan University of Medical Sciences and the informed consent of the elderly, the subjects entered the study voluntarily, and all the information remained confidential. The study samples were evaluated by the tools described below.

**Mini-mental state examination (MMSE):** One of the most common tools for measuring cognitive issues is the mini-mental state examination. Its advantages are its low cost, high speed, easy portability, and the ability to run and interpret with brief training. This instrument was designed by Folstein et al. in 1975. The results showed that MMSE, given the overall cut-off score (21), can differentiate normal subjects from patients with dementia at a sensitivity of 95% and specificity of 97%. The cut-off point was also calculated for male (18) and female (17) subjects separately. The test-retest reliability results revealed after ten days that the MMSE test had a reliability of 0.73. The test validity indices of MMSE (sensitivity 95%, false negative 5%, false positive 3%, and an overall cut-off point of 21) represent its desirable characteristics. This questionnaire provides information about spatial and temporal orientation, recording new data, attention and calculation, remembrance, language skills, 3D skills, and executive functioning. The maximum score in this test is 30, a score above 22-24 stands for mild cognitive impairment, a score of 10 to 21 signifies moderate cognitive impairment, and a score below 10 represents severe cognitive impairment. This test has been standardized for assessing the cognitive health of Iranian older adults [15-16)]. Hui Linet al.’s study showed that the MMSE score had good internal consistency, the value of Cronbach’s alpha coefficient being 0.82 [17].

**Lawton Instrumental Activities of Living Scale (IADL):** To analyze the functional ability of the elderly, the following items were considered: using the mobile phone, buying daily necessities, cooking, cleaning the house, washing clothes, using transportation services, using drugs, and managing finances. The maximum score on this test is 16. Sensitivity and specificity were 0.75 and 0.96, respectively; besides, Cronbach’s alpha and the intraclass correlation exceeded 0.75 [18].

**Katz Activities of Daily Living Scale:** The criteria for this test include: eating, wearing clothes, bathing, moving around the house, going to the bathroom, and bladder and bowel continence. Code one suggests the failure in doing a task, and a zero code implies the absence of such a defect. The range of answers to the questions will be between 0-6 for ADL and 0-9 in the case of IADL. Sensitivity and specificity of this test were 0.71 and 0.77, respectively. Cronbach’s alpha was above 0.75 [18].

**Stanford health assessment questionnaire 8-item disability index (HAQ 8-item DI):** The complete version of HAQ, including five sub-scales, was devised in 1978 by James F. Fries et al. at Stanford University. One of the subscales of this instrument is the disability scale (HAQ-DI) which has been frequently employed as an independent questionnaire. This scale, measuring one’s functional abilities, is referred to as HAQ in many studies. The questionnaire contains a total of 20 questions. Disability has eight dimensions, each including 2 to 3 questions. The tool measures the degree of a person’s disability on a scale of 0-3 so that a higher score indicates a more significant disability. So far, it has been translated into several languages, and numerous researchers have either investigated or used it in different countries. This form has eight questions related to the ability to dress up, go to bed and get up, lift a cup or glass to the mouth, walk on a smooth surface, wash and dry the whole body, bend and gather clothes from the ground, open and close water taps, and get into and out of the car. Cohen’s kappa coefficient in relation to each item was reported to be good or excellent (Kappa > 0.7), and the intraclass correlation coefficient for the whole instrument was 0.992. The coefficient of internal stability was estimated at 0.95-0.98. Calculating the correlation of each question with the total score of the questionnaire substantiated the reliability of the instrument [19].

**Inclusion and exclusion criteria**

**Inclusion Criteria:** At least 60 years of age and no history of psychiatric illness until test implementation.

**Exclusion Criteria:** Unwillingness to cooperate during the interview and completing the questionnaire in the middle of the program; failing to understand the interviewer’s questions; a disturbance in intellectual functioning or any symptom that indicates a lack of understanding of the questions; having a verbal-auditory impairment.

## Results

Table 1 shows the demographic characteristics of 393 individuals in the research samples.

Table 2 illustrates the prevalence of cognitive impairment in Gilan Province, based on MMSE cutting points.

Table 3 shows the prevalence of cognitive impairment in terms of ADL and IADL dependency among the older adults in Guilan Province. The Chi-square test revealed that there is a significant correlation (P = 0.001) between cognitive impairment (assessed based on the MMSE questionnaire) and the scores of ADL (121.68) and IADL (149.08).

**Table 1:** Demographic characteristics of research samples

	Variables	Number	Percentage
Gender	Female	201	51.1
	Male	192	48.9
Age	60-64	147	37.4
	65-69	100	25.4
	+70	146	37.2
Education	Below High School Diploma	330	84
	High School Diploma and Higher	63	16
Marital Status	Currently Married	285	72.5
	Currently Single	106	27
	Never married	2	0.5
Occupational Status	Employed	83	21.1
	Unemployed	121	30.8
	Retired	25	6.4
	Housewife	164	41.7

**Table 2:** Relative frequency of cognitive impairment among the older adults in Gilan Province

	Variables	Number	Percentage
Cognitive impairment	Severe cognitive impairment	17	4.3
	Moderate cognitive impairment	112	28.6
	Mild cognitive impairment	145	37
	Healthy	119	30.1
	Total	393	100

Table 4 presents the relative frequency of cognitive impairment in terms of demographic characteristics among seniors in Guilan Province. Chi-square test suggested that cognitive impairment is higher in women than men; additionally, people over 70 years of age had more severe cognitive impairment compared to other age groups.

In this study, the prevalence of disability for the elderly in Guilan province was 84.1%. Pearson correlation analysis showed that there is a significant relationship between elderly disability and cognitive impairment. As cognitive impairment intensifies in the seniors, their disability also increases (P-value = 0.05).

Table 5, drawing on multiple linear regression, illustrates the results of multiple analysis of the factors associated with cognitive impairment in the elderly. The results exhibited that in the final model of regression, which was run stepwise with the probability of entering (0.05) and removing (0.01) the variables, the two variables of IADL (P <0.001) and ADL (P <0.05) had the most significant relationship with cognitive impairment.

**Table 3:** Frequency of cognitive impairment in terms of ADL and IADL dependency among elderly people in Gilan Province.

		Cognitive impairment				
		Severe	Moderate	Mild		
ADL	Yes	5	83	107	Significance level (P= 0.001)	X2 = 121.68
	No	12	29	38		
IADL	Yes	10	91	123	Significance level (P= 0.001)	X2 = 149.08
	No	7	21	22		

**Table 4:** Relative frequency of cognitive impairment in terms of demographic characteristics in the older adults in Guilan Province

	N = 393	Cognitive impairment				P-value
		Score	Mild	Moderate	Severe	
Gender						0.001
Male	192	41.7	17.7	0.37	3.6	
Female	201	19.4	16.4	59.7	4.5	
Age						0.01
60-64 years	147	0.34	14.3	47.6	4.1	
65-69 years	100	0.32	0.25	0.38	0.5	
≤70 years	146	25.3	14.4	56.9	3.4	
Education						0.001
<12 years	330	22.7	16.4	56.4	4.5	
≥ 12 years	63	69.8	20.6	7.9	1.7	

**Table 5:** Multiple analysis of factors related to the elderly with cognitive impairments

Predictive factors of cognitive impairment		Non-standardized coefficients		Standardized regression coefficient	P value (Significance level)	Trust distance 0.95 Regression coefficient	
		Regression coefficient	Standard error			Lower bound	Upper bound
1	Fixed value (effect of unknown factors)	3.392	0.150	—	0.001	3.097	3.686
	IADL	- 0.061	0.013	- 0.231	0.001	- 0.088	- 0.035
2	Fixed value (effect of unknown factors)	2.084	0.315	—	0.001	1.465	2.704
	IADL	- 0.072	0.015	- 0.271	0.001	- 0.101	- 0.042
	ADL	0.080	0.032	0.140	0.05	0.017	0.143

## Discussion

Aging is an ongoing process, and it is only by having a healthy life and proper care that old age can be turned into a desirable and salubrious period. Although mental pressures are part of the everyday lives of older adults, unpleasant things are often beyond their control and they are usually accompanied by a decline in cognitive functioning and other disorders, it is still possible to reduce their fast progression with timely detection and control some of their associated disorders. Cognitive impairments in the elderly lead to a fall in the quality of life, loss of efficiency, decrease in one’s useful life, and ultimately an increase in mortality rates.

Our study aimed to investigate the prevalence of cognitive impairment and its effect on the performance of elderly people living in Guilan Province. The results suggest that 4.3%, 28.6%, and 37% of the study subjects had a severe, moderate, and mild cognitive impairment, respectively. Overall, 70% of the seniors in Guilan suffered from cognitive impairment. Our results are compatible with those of Kazemi who reported the incidence of severe cognitive impairment to be 5% and mild and moderate cognitive impairments to be 63% among their subjects [20]. Similarly, Mirzaee et al. have reported a 74.3% prevalence of cognitive impairment in the eldery of Khorramabad city [21]. However, the findings of the current research are not in line with those observed by Kheirkhah who reported that the prevalence of cognitive impairment is 31.6% in Amirkalayeh city. It should be noted that the time at which Mirzaee’s study was conducted is closer to the present study compared to that of Kheirkhah. This can be an alarm for health officials to do a national survey in this regard [5].

In the USA, the estimated prevalence of Cognitive Impairment, No Dementia after 70 years of age was 22.2%, ranging from 16% (71–79 years) to 39% (≥90 years) [22]. In some reviews, this prevalence ranges from 5 to 29% [23]. Among African-Americans, the prevalence was 23.4% (increasing with age, from 19.2% to 38%) [24]. A study in Taiwan observed a prevalence of 9.7% with a great influence from schooling [25].

Studies carried out on seniors in Ukraine, Taiwan, India, Portugal, Spain, Malaysia, and North Korea between 2005 and 2012 have reported the prevalence of cognitive impairments between 11% and 20% as assessed by MMSE (16-27). Kheirkhah et al., conducting a project on aging and health, estimated the prevalence of cognitive impairment at 18.3% [5]. Nevertheless, in the present study, the prevalence of cognitive impairment in Guilan was 32.9%. This amount is greater than those associated with other provinces in Iran, but it is somewhat consistent with the results of the research by Hatami et al. who observed that the prevalence of cognitive impairment was 46% [28]. With these impairments, attention, memory, language, orientation, actions, executive functioning, judgment, and problem-solving skills are disturbed. As age proceeds, mild impairment leads to dementia; therefore, a timely screening and early detection and treatment can be of great assistance in this regard. The increasing trend of cognitive impairment in the elderly from Iran requires the immediate attention of health authorities.

Investigating the impact of cognitive impairment in terms of ADL and IADL variables on seniors implies that there is a significant relationship therein so that with the growth of cognitive impairment, the ability of seniors significantly decreases. Consequently, these individuals lose their autonomy and become dependent on others to do their daily routines. This dependency is greater in severe and moderate cases. These results are in line with the findings of Bombin et al. [29] and De Vriendt et al. in Belgium [13].

The onset of the decline in ADL and IADL in our study sharply increased in the age group of subjects over 70 years. This suggestion could be confirmed by the longitudinal study of Jacobs et al., which was carried out over 20 years in Jerusalem [30] and also by the study of Perneczky et al., which proposed a strong correlation between the cognitive status and the ability to perform daily tasks [31]. The results regarding neuropsychological tests (e.g., working memory, delayed recall memory, and executive function) are consistent with those of Albert, Moss, Tanzi, and Jones [32], who reported that verbal and auditory memory tasks, the Part B of the Trail Making Test for executive function, and the backward digit span test were all indices of progression from health to mild Alzheimer’s Disease. The study was novel in that it demonstrated the ways in which the effects of cognitive function on IADLs differed according to IADL category, suggesting that certain cognitive functions could be prioritized during IADL-related rehabilitation.

In the present study, there was a significant difference between the two sexes concerning cognitive impairment, so that it was higher in women than in men. Furthermore, cognitive impairment was more significant in subjects over 70 years of age than others below this age. This is consistent with the report of Kheirkhah et al. [5], but incompatible with the study of Hatami et al., which was performed in 2014 on the rural elderly in Markazi province [24] because the latter did not observe a significant difference between men and women in terms of the prevalence of cognitive impairment. The results of the present study demonstrated that there is no significant difference between cognitive impairment and place of residence (city/village). The environment, therefore, does not seem to play a role in cognitive impairment. Inasmuch as previous studies have not considered the possible effect of urban or rural life on the results, the generalization of the above suggestion is open to further inquiry.

In the present study, a significant difference was observed between education and cognitive impairment. This is consistent with the results of the studies by Nejati in Iran [33, 34] and Rashid in Malaysia [35]. Meta-analyses have correlated low educational level with a higher risk for dementia [36]. A systematic review pointed out that education is not uniformly described as a risk factor for dementia in all studies. 61% of 46 studies had described a significant risk in the case of a low education level for developing dementia. In 9 studies from Latin America, education and dementia risk were linked [37].

In the multiple analysis of factors associated with cognitive impairment in the elderly, it was shown that in the final model, IADL and ADL variables had the most significant relationship with the cognitive impairment. Cognitive impairment has led to a decline in the performance of personal activities, which, in turn, can reduce the independence and quality of a senior’s healthy life. Consequently, many elderly need to be cared for after the emergence of cognitive impairment.

## Conclusion

In summary, CI represents a useful clinical entity. Most practitioners recognize persons in their clinical practices who meet the criteria for CI. The challenge is to help the clinicians revise their thresholds for detecting subtle cognitive impairments to enable them to identify persons with suspected CI. It is essential to realize that Mild Cognitive Impairment is a clinical diagnosis which is the same as are the diagnoses of dementia or Alzheimer’s Disease. While cognitive tests and functional measures are beneficial, ultimately, the final determination relies on the clinician’s judgment. The procedures outlined here were designed to assist in that process. As with other studies, this research has its limitations, one of which concerns those people who, due to their old age, were not able to visit health centers To overcome this problem, the mental health expert, with prior coordination, would refer to the selected rural health center.

It should be noted that all participants in this study were seniors who had an up-to-date record in the health centers. Thus, the study sample does not include all seniors from Guilan. Ultimately, the age distribution was not equally respected in the groups because according to the country’s latest census, the last population group is 64 years old or older. However, in this research, they were divided into two parts. The number of samples in the two classes should have been designed so that distribution would be closer to the average population. To maintain greater autonomy and better social participation, the authors recommend that seniors perform their daily activities themselves.

Additionally, doing daily activities has been recognized as an effective factor in preserving the health and success of the elderly population. Hence, efforts and programs aimed at maintaining and promoting the daily activities of old people are invaluable. Since the elderly population studied in this research was mostly literate, it is suggested that future studies should take into account the literacy distribution among the samples. Besides, it is fruitful to conduct an investigation concerning the relationship between cognitive impairment in the elderly and quality of life and happiness in other groups and other areas. It is also recommended that effective therapeutic interventions be employed to treat the cognitive impairment of seniors. Finally, a comprehensive national project is highly recommended to identify and address the health problems of the elderly.

## Acknowledgment

The authors feel obliged to express their gratitude to all the people who participated in this research.

## Conflict of Interest

The authors confirm that there are no conflicts of interest.
