# Correction to: An investigation into the prevalence of cognitive impairment and the performance of older adults in Guilan province

**Published:** 2018

**Authors:** Robabeh Soleimani, Somayeh Shokrgozar, Mahnaz Fallahi, Hashem Kafi, Maryam Kiani

**Affiliations:** 1. Psychiatry Department, Kavosh Cognitive Behaviour Sciences and Addiction Research Center, Shafa educational – remedial Hospital, Associate Professor, Guilan University of Medical Sciences, Rasht, Iran; 2. Psychiatry Department, Kavosh Cognitive Behaviour Sciences and Addiction Research Center, Shafa educational – remedial Hospital, Assistant Professor, Guilan University of Medical Sciences, Rasht, Iran; 3. Vice-chancellor for health, Guilan University of Medical Sciences, Rasht, Iran; 4. Guilan University of Medical Sciences, Rasht, Iran

## Abstract

In the original publication, the corresponding author name has been incorrectly typeset as: Robabeh Soleimani. The correct corresponding author name is: Somayeh Shokrgozar. The original article is now corrected.

In the original publication J Med Life 2018; 11:247-53[1], the corresponding author name has been incorrectly typeset. The correct corresponding author name is: Somayeh Shokrgozar. The original article is now corrected. We wish to apologise for any misunderstanding or inconvenience caused.
